# Compensatory enhancement of orexinergic system functionality induced by amyloid-β protein: a neuroprotective response in Alzheimer’s disease

**DOI:** 10.3389/fphys.2025.1529981

**Published:** 2025-03-24

**Authors:** Chenyu Zhuang, Hengyu Yan, Jiayu Lu, Yifan Zhou, Yanqing Liu, Guoshan Shi, Yan Li

**Affiliations:** ^1^ Medical College, Yangzhou University, Yangzhou, China; ^2^ The Key Laboratory of Syndrome Differentiation and Treatment of Gastric Cancer of the State Administration of Traditional Chinese Medicine, Yangzhou, China; ^3^ Department of Basic Medical Sciences, Guizhou University of Chinese Medicine, Guiyang, China; ^4^ Jiangsu Key Laboratory of Integrated Traditional Chinese and Western Medicine for Prevention and Treatment of Senile Diseases, Medical College of Yangzhou University, Yangzhou, China; ^5^ Department of Traditional Chinese Medicine, Affiliated Hospital of Yangzhou University, Yangzhou, China

**Keywords:** Alzheimer’s disease, orexin (hypocretin), amyloid-β protein, cognitive impairment, neuroprotection

## Abstract

**Background:**

Amyloid-β protein (Aβ) accumulation is a defining characteristic of Alzheimer’s disease (AD), resulting in neurodegeneration and a decline in cognitive function. Given orexin’s well-documented role in enhancing memory and cognition, this study investigates its potential to regulate Aβ-induced neurotoxicity, offering new perspectives into AD management.

**Methods:**

This paper simulated Aβ accumulation in the hippocampus of AD patients by administering Aβ_1-42_ oligomers into the bilateral hippocampal dentate gyrus of ICR mice. Inflammatory cytokines (IL-6, TNF-α) and orexin-A levels were measured by ELISA. Additionally, the excitability of orexinergic neurons was assessed by IHC targeting c-Fos expression. These methodologies evaluated the Aβ-induced neuroinflammation, orexinergic system functionality, and dexamethasone’s (Dex) effects on these processes.

**Results:**

Injection of Aβ_1-42_ oligomer resulted in elevated levels of IL-6, TNF-α, and orexin-A in the hippocampus, as well as increased excitability of orexinergic neurons in the lateral hypothalamus (LH). Dex treatment reduced neuroinflammation, causing a reduction in orexin-A levels and the excitability of orexinergic neurons.

**Conclusion:**

Aβ-induced neuroinflammation is accompanied by enhanced levels of orexin-A and orexinergic neuron excitability. These findings suggest that the enhanced functionality of the orexinergic system may become a compensatory neuroprotective mechanism to counteract neuroinflammation and enhance cognitive function.

## 1 Introduction

Alzheimer’s Disease (AD) is the most prevalent form of dementia, posing a significant challenge and having a profound impact on the health and quality of life of the elderly population. In recent years, China has witnessed an increase in the incidence, prevalence, and mortality rates of AD, imposing a considerable economic burden on patients’ families, society, and the healthcare system (Li et al., 2022; Ren et al., 2022; Ji et al., 2024). Despite advancements in diagnostic techniques and a deepening understanding of its pathology, the lack of a clearly defined underlying mechanism has hindered the development of effective treatments to slow the progression of AD. Pathologically, AD is featured by substantial atrophy and death of brain cells. The primary cause of this degeneration is the accumulation of extracellular amyloid-β protein (Aβ) plaques, coupled with intraneuronal neurofibrillary tangles (NFTs) composed of hyperphosphorylated tau proteins (Braak and Braak, 1991; Winblad et al., 2016). The presence of Aβ plaques and NFTs disrupts neuronal communication, resulting in synaptic loss and ultimately causing widespread neuronal death (Selkoe, 2002; Mattson, 2004). Further pathological features include gliosis, neuroinflammation, and significant synaptic changes (Itagaki et al., 1989; Calsolaro and Edison, 2016; Tönnies and Trushina, 2017).

Orexin, also referred to as hypocretin, is a neuropeptide synthesized in the hypothalamus and consists of two isoforms: orexin-A and orexin-B (de Lecea et al., 1998; Sakurai et al., 1998). Orexinergic neurons are primarily located in the lateral hypothalamus (LH) and extensively project their fibers to different regions of the brain (Vraka et al., 2023). The orexinergic system is essential in regulating sleep-wake cycles, feeding behavior, energy homeostasis, as well as reward processing (Chemelli et al., 1999; Edwards et al., 1999; Volkoff et al., 1999; Willie et al., 2001; Carter et al., 2009; Tsujino and Sakurai, 2009; Leinninger et al., 2011). Extensive research has demonstrated that the orexinergic system exerts an influence on the pathophysiology of AD, particularly in relation to the generation and accumulation of Aβ. Kang et al. discovered that injecting orexin into the ventricles of APP/PS1 mice elevates the levels of Aβ in the brain ISF, while infusion of a dual orexin receptor antagonist (DORA) effectively decreases Aβ levels (Kang et al., 2009). Similarly, studies have indicated that knocking out the orexin gene in APP/PS1 mice results in a reduction of Aβ deposition in the brain (Roh et al., 2014). Human studies have revealed that patients with moderate to severe AD present higher levels of orexin in their CSF compared to the control individuals (Liguori et al., 2014). However, there remains some disagreement regarding whether the orexinergic system actually promotes the progression of AD. On one hand, focal overexpression of orexin in the hippocampus of APP/PS1/OR^−/−^ mice did not alter the levels of Aβ accumulation (Roh et al., 2014). Additionally, *postmortem* analysis of hypothalamic tissue from patients with AD displayed a 40% reduction in orexin-immunoreactive neurons, accompanied by a slight decrease in the ventricular cerebrospinal fluid (CSF) levels of orexin-A. (Fronczek et al., 2012).

On the other hand, some physiological functions of orexin are inconsistent with a role in the development of AD. Orexin plays a crucial role in hippocampus-dependent social memory consolidation, and the introduction of exogenous orexin can ameliorate this deficit by modulating synaptic plasticity in the hippocampal region (Yang et al., 2013). By regulating the activity of various neurotransmitter systems, including cholinergic and dopaminergic pathways, orexin can enhance learning processes, facilitate the retrieval of memories, as well as support the consolidation of acquired information (Telegdy and Adamik, 2002; Palotai et al., 2014; Piantadosi et al., 2015). The administration of orexin-A via the nasal cavity or directly into the hippocampus has been shown to alleviate memory deficits in orexin/ataxin-3 transgenic mice (Yang et al., 2013; Mavanji et al., 2017). The impairment of memory caused by orexin deficiency has been confirmed in humans. For instance, patients with narcolepsy frequently exhibit memory and cognitive decline that is associated with the disease (Henry et al., 1993; Naumann et al., 2006; Witt et al., 2018). Other studies have demonstrated that orexin exerts neuroprotective effects. Specifically, orexin-A inhibits neuroinflammation by decreasing astrocyte proliferation, microglial activation, as well as the expression of chemokines and cytokines (Becquet et al., 2019). Orexin receptors hinder excessive autophagy through the MAPK/ERK/mTOR signaling pathway and confer neuroprotective benefits in AD via heterodimerization with GPR103 (Davies et al., 2015; Xu et al., 2021).

In summary, considering the diverse physiological functions of orexin, it is unreasonable to regard it as a direct causal factor in AD. The function of orexin in bolstering cognitive abilities, particularly memory and learning, fundamentally contradicts the underlying pathological mechanisms of AD. Although some studies suggest that orexin influences Aβ dynamics, its overall beneficial effects indicate a protective, rather than pathogenic, role in AD. Therefore, we hypothesize that the increased orexin levels observed in the brains of AD patients may represent a compensatory neuroprotective mechanism, rather than a causative factor. The accumulation of Aβ stimulates a compensatory increase in orexin, which, in turn, enhances a range of neuroprotective mechanisms, thereby mitigating the memory and cognitive impairments related to Aβ deposition. To certify this hypothesis, the pathological characteristics of Aβ accumulation in the hippocampus of AD patients were simulated in this study by injecting Aβ_1-42_ oligomers into the bilateral hippocampal dentate gyrus. Subsequently, we observed the impact of various concentrations of Aβ_1-42_ oligomers on neuroinflammation in the hippocampus and the functionality of the orexinergic system in the brain. Additionally, the intervention effects of dexamethasone (Dex) on these impacts were evaluated.

## 2 Materials and methods

### 2.1 Experimental reagents and main apparatus

Human Amyloid Peptide (1–42) (P9001, Beyotime Biotechnology, Shanghai, China) was dissolved in dimethyl sulfoxide (DMSO; ST038, Beyotime Biotechnology, Shanghai, China) to prepare a 3 mM stock solution. This solution was then diluted in sterile phosphate-buffered saline (PBS; PYG0021, Boster Biological Technology, Wuhan, China) to achieve the desired concentration for Aβ oligomers formation. Dexamethasone sodium phosphate solution (Hefei Zhonglong Shenli Animal Pharmaceutical Co., Ltd., Hefei, China) was administered to specific mouse groups to investigate its effects on neuroinflammation and orexin-A expression. Injections were performed using a Mouse Brain Stereotaxic apparatus (STW-3, Chengdu Instrument Factory, Chengdu, China) and Microliter Syringes (PYG0021, Shanghai High Pigeon Industry & Trade, Shanghai, China). The injection rate was controlled by a Dual-channel Intelligent Syringe Pump (XMSP-2B, Ximai Nanotech, Shanghai, China). Quantification of IL-6, TNF-α, and orexin-A levels in hippocampal tissues was performed using Mouse IL-6, TNF-α, and orexin-A ELISA Kits (BYabscience, Nanjing, China; Catalog Numbers: BY-EM220188, BY-EM220852, BY-EM228148). Immunohistochemical analysis to assess orexinergic neuron excitability used an Anti-c-Fos antibody (ab222699, Abcam, Cambridge, United Kingdom) and the SABC-POD (F) rabbit IgG kit (SA1028, Boster Biological Technology, Wuhan, China) to detect c-Fos protein expression.

### 2.2 Prepare Aβ oligomers

Drawing on prior reports on preparing Aβ oligomers (Kim et al., 2016), we made a 3 mM stock of Aβ_1-42_ in dimethyl sulfoxide (DMSO) and then diluted it 10-fold in phosphate-buffered saline (PBS) (300 *μ*M Aβ, 90% PBS, 10% DMSO). The Aβ_1-42_ monomer solution was then incubated at 37°C for 24 h to promote the formation of oligomers. After incubation, the solution was dispensed and frozen at −80°C for later use.

### 2.3 Animals

Male ICR mice (8–10 weeks old, weight 25 ± 3 g) were sourced from the Comparative Medicine Center of Yangzhou University (Yangzhou, China). These mice were housed in standard laboratory conditions, with a temperature maintained at 25°C ± 1°C, humidity levels kept between 45% and 50%, and a controlled light cycle from 6 a.m. to 6 p.m. The animal protocol followed the ethical guidelines and scientific standards approved by the Institutional Animal Care and Use Committee of Yangzhou University (ethical approval code: YXYLL-2024-111, date: 21 May 2024). The animals were randomly assigned to seven groups: a Dex group, a sham group, a low-concentration Aβ_1-42_ group, a medium-concentration Aβ_1-42_ group, a high-concentration Aβ_1-42_ group, a high-concentration Aβ_1-42_ + Dex group, as well as sham + Dex group, with 6 animals in each group.

**FIGURE 1:**
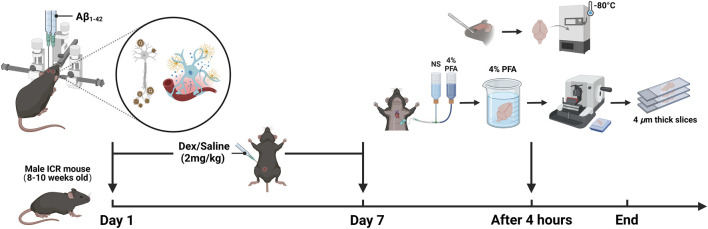
The design of experiments (created with BioRender.com).

The sham group received an injection of 1 μL of 0.9% saline solution into the bilateral hippocampal dentate gyrus. The groups receiving low, medium, and high concentrations of Aβ_1-42_ oligomers were injected with 125 μM, 250 μM, and 375 μM of the oligomers, respectively, all at the same volume and injection site. For seven consecutive days, the Dex group, sham + Dex group and the high-concentration Aβ_1-42_ + Dex group underwent daily intraperitoneal injections of Dex (2 mg/kg). In contrast, the remaining groups received an equivalent volume of saline. On the seventh day, 4 hours following the intraperitoneal injection, the brains of the mice were collected for further analysis. For the purpose of conducting an enzyme-linked immunosorbent assay (ELISA), the brain was promptly excised, snap-frozen, and then stored at −80°C to ensure the preservation of its biological activity. For immunohistochemistry (IHC) analysis, the mice were anesthetized and underwent transcardial perfusion with 0.9% saline, followed by 4% paraformaldehyde (PFA). Subsequently, the brains were extracted and immersed in 4% PFA overnight for fixation. Following fixation, the brains were dehydrated, embedded in paraffin blocks, and then sectioned into 4 μm sagittal paramedian slices using a microtome. The sections were positioned between 0.7 and 0.74 mm lateral to the brain midline, which corresponds to the location of the LH. For each brain tissue, three consecutive sections were retained, and the average ratio of the positive area from these sections was computed to serve as the representative value for each individual sample (as illustrated in Figure 1).

### 2.4 Bilateral intrahippocampal injection of Aβ_1-42_ oligomers in mice

The mice are first weighed and then anesthetized using 1% pentobarbital sodium (50 mg/kg) via intraperitoneal injection. Adequacy of anesthesia is confirmed by assessing the absence of a response to a pinch on the tail tip. Subsequently, the hair on the head of each mouse is cut off, and the mouse is fixed onto the ear bar of the brain stereotaxic apparatus. To prevent corneal dryness, sterile PBS is instilled into both eyes. The skin of the head is disinfected with iodophor, and a 1 cm incision is made in the center using ophthalmic scissors. The incision is then put open, and the mucosa covering the skull surface is peeled away using two injection needles, which help to dry the skull surface and facilitate the identification of the bregma. Once the bregma is identified, a point 2.0 mm posterior and 1.4 mm lateral to it is marked. At this marked location, a hole with a diameter of 1 mm is created using a grinder. Different concentrations of Aβ_1−42_ oligomers are drawn into a bilateral microsyringe and fixed onto the brain stereotaxic instrument. The needle tip is then moved to the drilled hole and inserted 2.5 mm from the skull surface to reach the hippocampus dentate gyrus. Aβ is injected simultaneously into both hippocampi over 5 min at a rate of 0.2 μL/min. Following the injection, the needle is left in place for an additional 5 min to aid in the diffusion of Aβ. The injection needle is then withdrawn, and the incision is sutured closed. After the surgery, each mouse is placed in a recovery box at 29°C until it has fully regained consciousness. During the first 3 days post-surgery, each mouse receives a daily subcutaneous injection of acetaminophen (200 mg/kg) for pain relief and enrofloxacin (5 mg/kg) for infection prevention, while closely monitoring the wound healing and overall health. If necessary, sterile PBS is applied to the wound area to maintain cleanliness. During the recovery period, the mice are housed individually in clean cages to prevent interference from other animals.

### 2.5 Enzyme-linked immunosorbent assay (ELISA)

The mouse ELISA kit should be removed from the refrigerator and allowed to equilibrate at room temperature for 30 min. The steps outlined in the kit manual should be strictly adhered to, using a microplate reader to ascertain the optical density (OD) values for IL-6, TNF-α, and orexin in mouse hippocampal tissue samples. Subsequently, standard linear regression curves should be plotted, with the horizontal coordinate representing the standard concentration of IL-6, TNF-α, and orexin, and the vertical coordinate corresponding to the respective OD values. Based on the curve equation derived, the concentrations of these biomarkers in the mouse hippocampal samples can then be calculated.

### 2.6 Immunohistochemistry (IHC)

The paraffin-embedded sections of brain tissue underwent deparaffinized in xylenes and were subsequently rehydrated through a graded series of alcohol solutions. After a 15-min pretreatment with 3% H_2_O_2_ to inactivate endogenous peroxidase activity, antigen retrieval was performed using citrate buffer. The sections were subsequently blocked with 5% BSA for 20 min and then incubated at 4°C overnight with the primary anti-c-Fos protein (1:500 dilution). The slices were then incubated with a secondary antibody for 30 min at 37°C, followed by the application of streptavidin-biotin complex (SABC) droplets. Between each step, the sections were thoroughly rinsed 3 times in Tris Buffered Saline (TBS) for 5 min each. The sections were then developed using diaminobenzidine (DAB) and counterstained with hematoxylin. Lastly, the sections underwent washing, dehydration, and clearing, as well as were mounted with cover slips.

Images of the sections were captured utilizing NIS Elements F 3.0 software (Nikon Corporation, Tokyo, Japan). Before photography, a blank site was evaluated with automatic white balancing. The immunohistochemical images were processed using ImageJ software (ImageJ 1.8.0, Rawak Software Inc., Stuttgart, Germany) to determine the ratio of positive areas.

### 2.7 Statistical analysis

Statistical analyses were conducted using GraphPad Prism version 10.1.2 (GraphPad Software, San Diego, CA, United States). The data for each group were presented as mean ± standard deviation (SD). One-way analysis of variance (ANOVA) was employed to compare data among multiple groups, followed by Tukey’s *post hoc* test for multiple comparisons. *P* < 0.05 was deemed statistically significant, whereas *P* > 0.05 was considered statistically non-significant (NS).

## 3 Results

### 3.1 Aβ_1-42_ oligomers induce an increase in the secretion of pro-inflammatory cytokines

**FIGURE 2:**
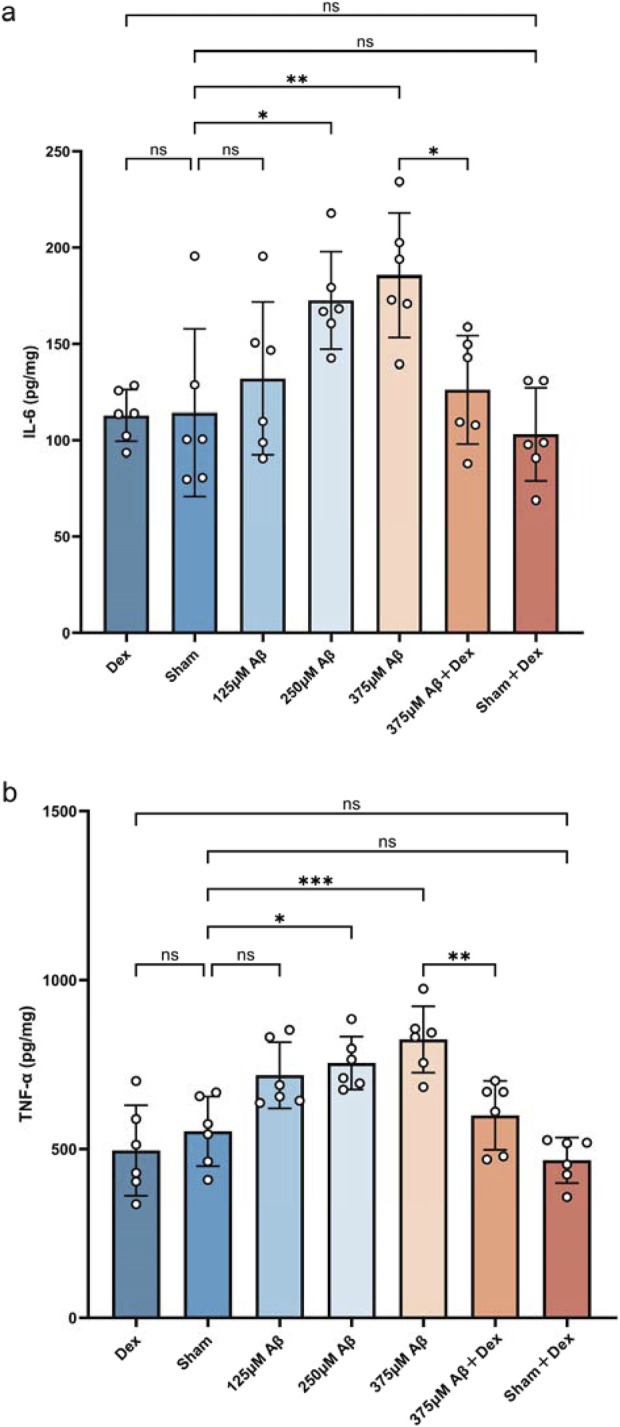
Aβ_1-42_ oligomers induce an increase in the secretion of pro-inflammatory cytokines. ELISA was utilized to measure the concentrations of inflammatory cytokines in hippocampal extracts obtained from each group. Injections of Aβ oligomers led to an upregulation in the expression of IL-6 **(A)** and TNF-α **(B)**, while Dex treatment significantly decreased their levels. Statistical analysis was performed using one-way ANOVA followed by Tukey’s test. For IL-6, the ANOVA F = 6.250, with Tukey’s *post hoc* test showing *p* > 0.9999 for Dex (112.9 ± 13.39, n = 6) vs. Sham (114.3 ± 43.64, n = 6), *p* = 0.9977 for Dex vs. Sham + Dex (103.1 ± 24.17, n = 6), *p* = 0.9524 for Sham vs. 125 μM Aβ (132.1 ± 39.71, n = 6), *p* = 0.0365 for Sham vs. 250 μM Aβ (172.6 ± 25.24, n = 6), *p* = 0.0054 for Sham vs. 375 μM Aβ (185.7 ± 32.32, n = 6), *p* = 0.9954 for Sham vs Sham + Dex, and *p* = 0.0309 for 375 μM Aβ vs. 375 μM Aβ + Dex (126.2 ± 28.16, n = 6). For TNF-α, the ANOVA F = 11.44, with Tukey’s *post hoc* test showing *p* = 0.9532 for Dex (495.4 ± 133.8, n = 6) vs. Sham (552.1 ± 103.1, n = 6), *p* = 0.9986 for Dex vs. Sham + Dex (466.4 ± 67.31, n = 6), *p* = 0.0847 for Sham vs. 125 μM Aβ (717.9 ± 98.02, n = 6), *p* = 0.0185 for Sham vs. 250 μM Aβ (754.2 ± 78.35, n = 6), *p* = 0.0006 for Sham vs. 375 μM Aβ (824.1 ± 98.51, n = 6), *p* = 0.7457 for Sham vs. Sham + Dex, and *p* = 0.0066 for 375 μM Aβ vs. 375 μM Aβ + Dex (599.5 ± 102.0, n = 6). Data represent mean ± SD (n = 6); *: *p* < 0.05, **: *p* < 0.01, ***: *p* < 0.001, ****: *p* < 0.0001.

The levels of IL-6 and TNF-α in the hippocampus of mice were detected by ELISA to assess the expression of proinflammatory cytokines. Compared to the sham group, both IL-6 and TNF-α levels were significantly increased in the Aβ groups (*p* < 0.05, Figures 2A,B), indicating that injection of Aβ oligomers induces neuroinflammation. Dex treatment markedly decreased the Aβ-induced elevation of IL-6 and TNF-α levels (*p* < 0.05, Figures 2A,B), demonstrating its effectiveness in inhibiting pro-inflammatory cytokines and mitigating neuroinflammation in AD. No significant differences were observed in the levels of IL-6 and TNF-α among Dex, Sham, and Sham + Dex groups.

### 3.2 Elevated orexin-A levels induced by Aβ_1-42_ oligomer injection exhibited a clear concentration-dependency

**FIGURE 3:**
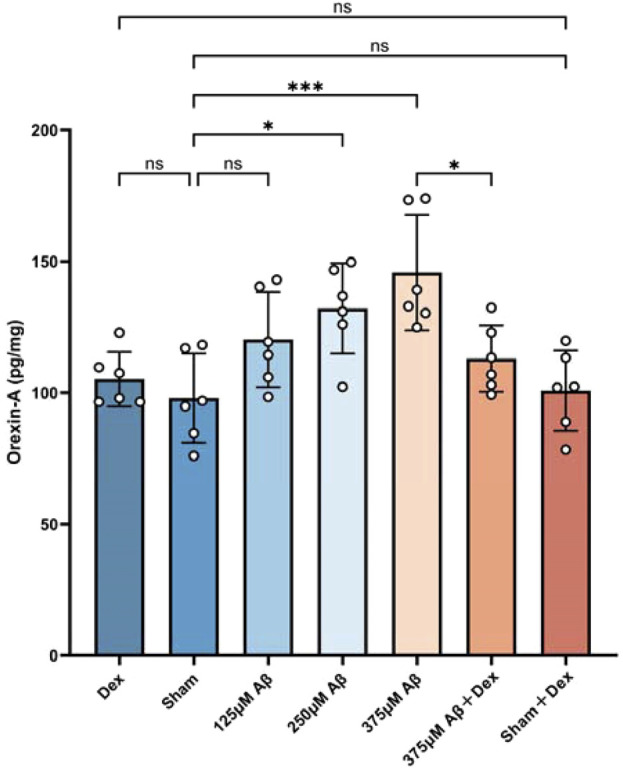
Elevated orexin-A levels induced by Aβ_1-42_ oligomer injection exhibited a clear concentration-dependency. The ELISA assay was used to evaluate the concentrations of orexin-A in hippocampal extracts obtained from different groups. The results demonstrated that injections of Aβ oligomers upregulated the expression of orexin-A **(c)**, while Dex treatment reduced its level. Statistical analysis was performed using one-way ANOVA followed by Tukey’s test. The ANOVA F = 6.797, with Tukey’s *post hoc* test showing *p* = 0.9875 for Dex (105.2 ± 10.41, n = 6) vs. Sham (97.95 ± 16.99, n = 6), *p* = 0.9992 for Dex vs. Sham + Dex (100.8 ± 15.31, n = 6), *p* = 0.2528 for Sham vs. 125 μM Aβ (120.2 ± 18.12, n = 6), *p* = 0.0158 for Sham vs. 250 μM Aβ (132.1 ± 17.19, n = 6), *p* = 0.0003 for Sham vs. 375 μM Aβ (145.8 ± 22.05, n = 6), *p* > 0.9999 for Sham vs. Sham + Dex, and *p* = 0.0230 for 375 μM Aβ vs. 375 μM Aβ + Dex (113 ± 12.65, n = 6). Data are presented as mean ± SD (n = 6); *: *p* < 0.05, **: *p* < 0.01, ***: *p* < 0.001, ****: *p* < 0.0001.

The levels of orexin-A in the hippocampus of mice continued to be monitored using the ELISA method. The results revealed that, compared to the sham group, orexin-A levels in the Aβ group were significantly evaluated (*p* < 0.05, Figure 3C), demonstrating a concentration-dependent relationship (*p* < 0.05, Figure 3C). Dex treatment significantly reduced orexin-A levels in the Aβ groups (*p* < 0.05, as displayed in Figure 3C), but the treatment had no significant effect on orexin-A levels in the sham + Dex group (*p* > 0.05, as shown in Figure 3C). No significant differences were observed in the levels of orexin-A among Dex, Sham, and Sham + Dex groups. This suggested that the increase of orexin-A was driven by the neuroinflammation induced by Aβ.

### 3.3 The excitability of orexinergic neurons in the lateral hypothalamus (LH) of AD mouse models induced by Aβ_1-42_ oligomers was increased

**FIGURE 4:**
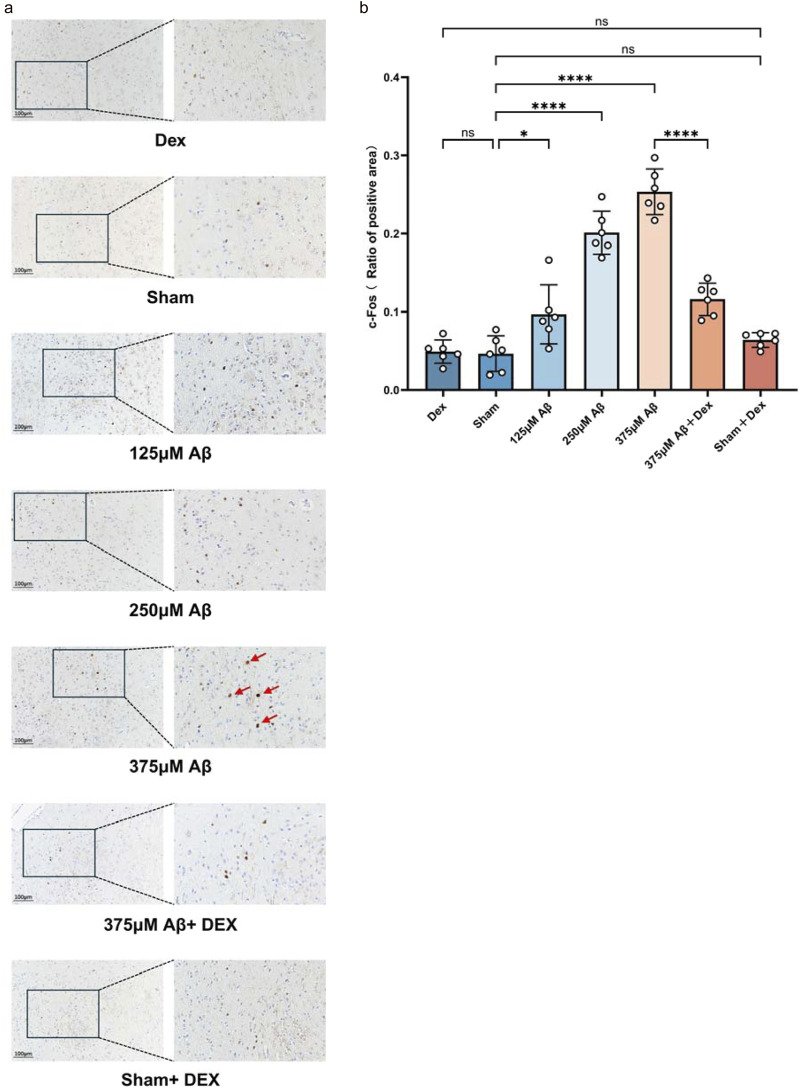
The excitability of orexinergic neurons in the lateral hypothalamus (LH) of AD mouse models induced by Aβ_1-42_ oligomers was increased. **(a)** Representative IHC staining images of c-Fos-positive neurons in the LH for each group, showing sagittal sections. Scale bar is indicated as 100 *μ*m. The enlarged view is provided for better clarity. The 200 μm view can be viewed in the supplementary files link (https://www.frontiersin.org/articles/10.3389/fphys.2025.1529981/full#supplementary-material). The quantitative analysis of the area positive for c-Fos in neurons, conducted using ImageJ software, is presented in **(b)**. The results demonstrated that the injection of Aβ oligomers increased c-Fos protein expression, resulting in an increased excitability of orexinergic neurons. Dex treatment specifically reduced the excitability of orexinergic neurons in the Aβ groups, without affecting the sham + Dex group. Statistical analysis was performed using one-way ANOVA followed by Tukey’s test. The ANOVA F = 62.8, with Tukey’s *post hoc* test showing *p* > 0.9999 for Dex (0.049 ± 0.01476, n = 6) vs. Sham (0.04633 ± 0.02271, n = 6), *p* = 0.9415 for Dex vs. Sham + Dex (0.06383 ± 0.009368, n = 6), *p* = 0.0189 for Sham vs. 125 μM Aβ (0.09667 ± 0.03789, n = 6), *p* < 0.0001 for Sham vs. 250 μM Aβ (0.2012 ± 0.02764, n = 6), *p* < 0.0001 for Sham vs. 375 μM Aβ (0.2533 ± 0.02904, n = 6), *p* = 0.8801 for Sham vs. Sham + Dex, and *p* < 0.0001 for 375 μM Aβ vs. 375 μM Aβ + Dex (0.1160 ± 0.02071, n = 6). Data are presented as mean ± SD (n = 6); *: *p* < 0.05, **: *p* < 0.01, ***: *p* < 0.001, ****: *p* < 0.0001.

Orexinergic neurons are predominantly located in the LH. To further assess functional changes in the orexinergic system, IHC was used to detect c-Fos positive neurons in the LH of mice. C-Fos gene, an immediate early gene transcribed in response to neuronal activation, is commonly used as a marker for identifying active neurons (Joo et al., 2016). The results demonstrated that compared to the sham group, injection of Aβ oligomer led to an increase in both c-Fos protein expression and the count of c-Fos-positive neurons (*p* < 0.05, Figures 4A,B), displaying a heightened excitability of orexinergic neurons. Additionally, the number of activated orexinergic neurons increased in correlation with higher concentrations of Aβ injection (*p* < 0.05, Figures 4A,B). Although Dex treatment significantly reduced c-Fos protein expression in the Aβ groups (*p* < 0.0001, Figures 4A,B), there was no measurable impact in the sham + Dex group (*p* > 0.05, Figures 4A,B), indicating that Dex does not affect the excitability of orexinergic neurons. No significant differences were observed in the count of c-Fos-positive neurons among the Dex, Sham, and Sham + Dex groups.

## 4 Discussion

Neuroinflammation is widely recognized as a central mechanism underlying the accumulation of amyloid-β protein (Aβ)-induced pathology in Alzheimer’s disease (AD). Research has shown that Aβ can stimulate microglia, causing them to shift towards the pro-inflammatory M1 phenotype and triggering the secretion of various pro-inflammatory factors, such as IL-6 and TNF-α, thereby initiating neuroinflammatory responses (Heneka et al., 2015; Tang and Le, 2016). This inflammatory response disrupts neuronal signal transmission, decreases synaptic plasticity, and enhances oxidative stress reactions, ultimately exacerbating neuronal damage (Block et al., 2007; Cai et al., 2019). Synaptic plasticity is a fundamental process that underlies learning and memory. The persistent release of pro-inflammatory factors can disrupt neuronal signaling pathways, causing synaptic loss and ultimately contributing to significant cognitive impairment in AD (De Strooper and Karran, 2016; Ransohoff, 2016). In this study, we successfully induced a significant upregulation of pro-inflammatory factors by injecting Aβ_1-42_ oligomers into the dentate gyrus of the mouse hippocampus, effectively simulating the neuroinflammatory pathological features of AD.

Previous studies have suggested that elevated orexin levels may lead to increased Aβ levels. For instance, Kang et al. found that injecting orexin into APP transgenic mice resulted in higher Aβ deposition, whereas administering a dual orexin receptor antagonist (DORA) reduced Aβ levels (Kang et al., 2009). Similarly, Roh et al. observed reduced Aβ deposition following the knockout of the orexin gene in APP/PS1 transgenic mice (Roh et al., 2014). These studies, along with our research, demonstrate a positive correlation between orexin and Aβ, although the causal relationships differ. It is important to note that the orexin system plays a key role in promoting and maintaining wakefulness (Arrigoni et al., 2010), facilitating voluntary activity (Kiwaki et al., 2004), and supporting energy metabolism (Tsujino and Sakurai, 2009). On one hand, during wakefulness, metabolic products such as adenosine and Aβ inevitably accumulate in the brain (Huang et al., 2011; Spira et al., 2014). On the other hand, there is literature showing that Aβ-degrading enzymes, such as neprilysin (NEP), exhibit increased activity during sleep when orexinergic neurons are inhibited, thereby enhancing Aβ degradation (Ogawa et al., 2005). However, previous studies seem to have overlooked the impact of metabolic product accumulation during wakefulness and the corresponding decrease in Aβ-degrading enzyme activity.

Recent research has demonstrated that the orexinergic system enhances cognitive functions. Specifically, orexin-A promotes the transformation of microglia into the anti-inflammatory M2 phenotype (Duffy et al., 2015), which exhibits anti-inflammatory and neuroprotective properties, improving cognitive deficits (Saijo and Glass, 2011; Wang et al., 2020). The role of the orexinergic system in regulating the body’s energy metabolism is well-established (Tsujino and Sakurai, 2009). From the survival perspective, it also fundamentally aids humans and animals in adapting to their environment and maintaining survival capabilities. For example, when animals are hungry or hypoglycemic, the excitability of orexinergic neurons increases (Risold et al., 1999), resulting in increased secretion of orexin-A (Ouedraogo et al., 2003). The enhanced functionality of the orexinergic system fosters and maintains wakefulness, augments autonomous activity, and boosts muscle energy consumption. Furthermore, it also enhances alertness and cognitive functions (Kiwaki et al., 2004; Yang et al., 2013). Collectively, these effects promote feeding behavior to fulfill energy requirements. This adaptive mechanism underscores the crucial role of the orexinergic system in ensuring survival by modulating physiological and behavioral responses according to the environment.

Neuroinflammation induced by the accumulation of Aβ results in cognitive impairments in both humans and animals, evidently compromising their adaptability and survival capabilities. To counteract the cognitive toxicity induced by Aβ, the body inevitably initiates compensatory responses. Our study demonstrates that intracerebral injection of Aβ led to a concentration-dependent increase in both orexin-A levels in the hippocampus, as well as an enhancement in the excitability of orexinergic neurons in the lateral hypothalamus (LH). This suggests that the accumulation of Aβ not only triggers neuroinflammation but also simultaneously augments the functionality of the orexinergic system.

Dexamethasone (Dex) is a glucocorticoid anti-inflammatory agent capable of penetrating the blood-brain barrier (BBB) (Vohra et al., 2021). In our study, intervention with Dex significantly mitigated Aβ-induced neuroinflammation, leading to decreased levels of orexin-A in the hippocampus and reduced excitability of orexinergic neurons in the LH. These findings further affirm that the elevation in orexin-A levels is a consequence of Aβ-induced neuroinflammation, and they bolster the hypothesis that the orexinergic system serves as a neuroprotective mechanism against Aβ-induced cognitive toxicity.

While our study provides valuable insights into the compensatory role of the orexinergic system in AD, there are several limitations that should be considered. First, although we focused on male mice to reduce variability caused by hormonal fluctuations, this choice limits our ability to fully assess potential sex-based differences in the impact of Aβ and the orexinergic system. Future studies should include both male and female animals to explore whether sex-based differences influence the compensatory mechanisms observed in AD. Furthermore, while our study utilized c-Fos as a marker of neuronal excitability, it is worth noting that more advanced techniques, such as electrophysiological recordings or calcium imaging, could offer a more comprehensive understanding of the dynamic activity of orexinergic neurons. This limitation could be addressed in future studies.

Besides, caution is needed when applying these findings to humans due to the inability of animal models to fully replicate the complexity of human AD, particularly in terms of genetics and the multifactorial nature of the disease. Further research is required to clarify the molecular mechanisms and signaling pathways through which the orexinergic system regulates AD pathology. Additionally, it is crucial to consider the implications of dosage, treatment duration, and long-term effects when exploring the orexinergic system as a therapeutic target. These considerations will help guide future research directions and their potential clinical applications.

The latest studies have explored various anti-inflammatory treatment strategies for AD. Both lupeol, a natural compound, and NB-02, a botanical therapeutic drug, have demonstrated neuroprotective effects by regulating neuroinflammation (Lee et al., 2021; Choe, 2024). Additionally, the activation of the receptor TREM2 on microglia has been proven to reduce neuroinflammation and improve cognitive outcomes in AD, while monoclonal antibodies targeting TREM2 further support its therapeutic potential (Fassler et al., 2021). Methylprednisolone and low-dose aspirin have also shown promise in regulating neuroinflammation and mitigating cognitive decline (Vallés et al., 2020; Sun et al., 2023). Furthermore, GLP-1 receptor agonists have been found to reduce neuroinflammation and amyloid precursor protein (APP) deposition, while improving memory and synaptic function (Nowell et al., 2022). In summary, these studies underscore the potential of targeting neuroinflammation as a therapeutic strategy for AD, and the findings of our study are in alignment with this growing body of work.

## 5 Conclusion

Neuroinflammation induced by Aβ is accompanied by an augmentation in the functionality of the orexinergic system, featured by an increase in the excitability of orexinergic neurons and an elevated secretion of orexin-A. The enhanced functionality of the orexinergic system acts as a compensatory response to counteract Aβ-induced neuroinflammation, ultimately aiming to improve cognition. This study contributes to our understanding of the pathophysiological mechanisms underlying AD.

## Data Availability

The original contributions presented in the study are included in the article/Supplementary Material, further inquiries can be directed to the corresponding author.

## Supplementary material

Supplementary Data Sheet S1 Representative IHC staining images of c-Fos-positive neurons in the LH for each experimental group, showing sagittal sections. The scale bars are indicated as 100 μm, 200 μm, and an enlarged view is provided for better clarity.
