# Supplementary figures and images for: Compensatory enhancement of orexinergic system functionality induced by amyloid-β protein: a neuroprotective response in Alzheimer’s disease
Source: Front Physiol. 2025 Mar 24;16:1529981. doi: 10.3389/fphys.2025.1529981 (PMC11973307; doi:10.3389/fphys.2025.1529981)

d

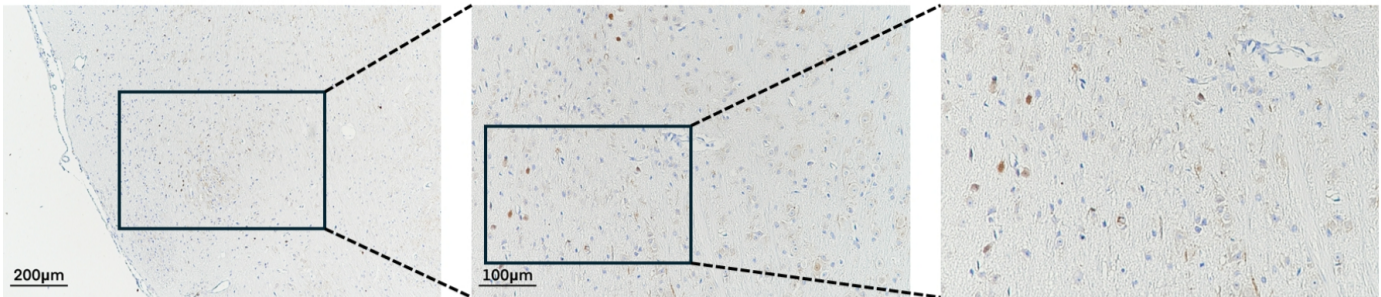

Dex

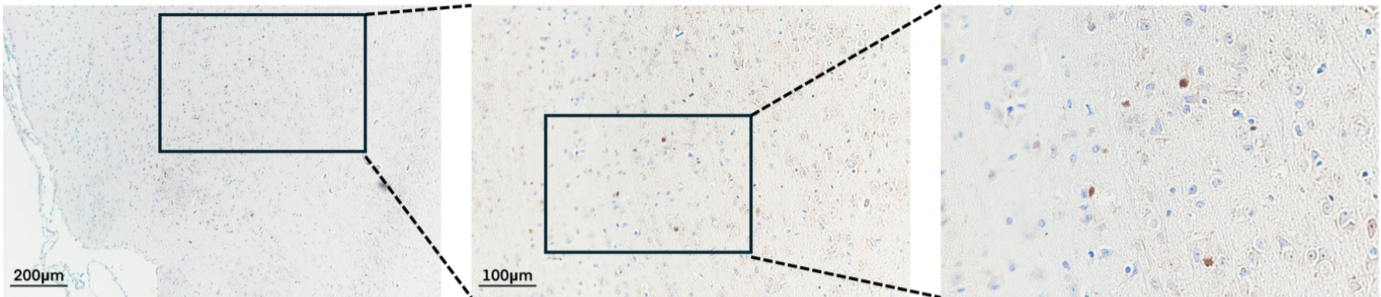

Sham

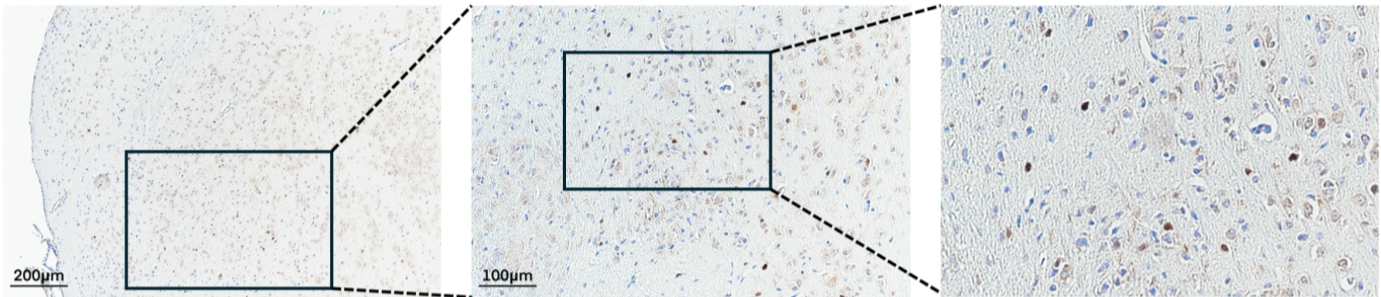

125µM Aβ

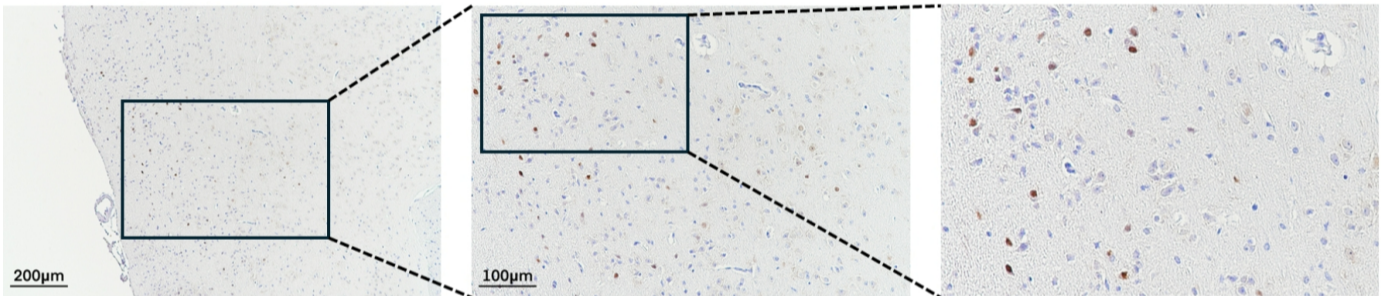

250µM Aβ

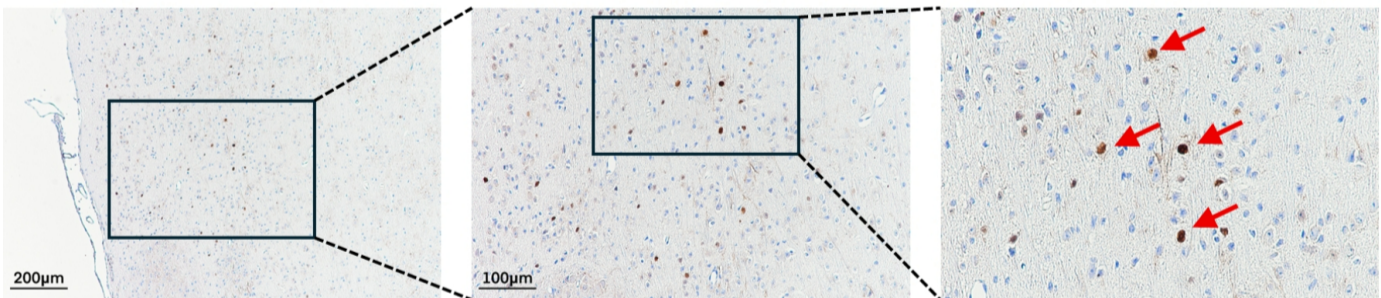

375µM Aβ

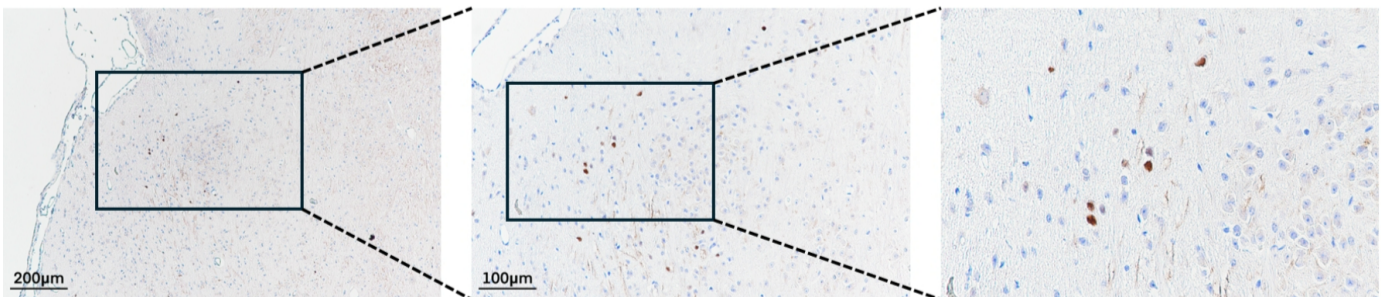

375µM Aβ+ DEX

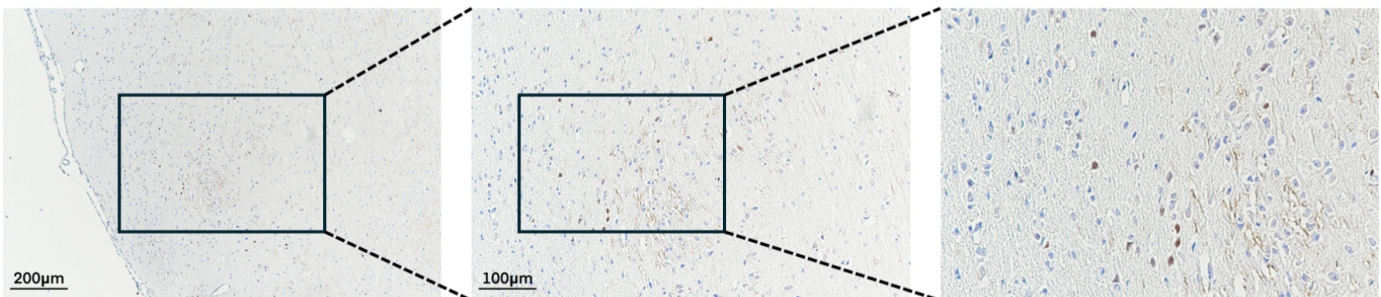

Sham+ DEX

Supplement: Supplementary file 1 [file DataSheet1.pdf]
